# Cost to play community-level sport in Australia

**DOI:** 10.3389/fspor.2026.1891152

**Published:** 2026-08-18

**Authors:** Rochelle Eime, Hans Westerbeek, Melanie Charity, Katherine Owen

**Affiliations:** 1 Sport and Physical Activity Insights, Institute of Health and Wellbeing, Federation University Australia, Ballarat, VIC, Australia; 2 Institute for Sustainable Industries and Liveable Cities, Victoria University Business School, Victoria University, Melbourne, VIC, Australia; 3 The University of Sydney Prevention Research Collaboration, The University of Sydney, Sydney, NSW, Australia

**Keywords:** affordability, community, cost, participation, sport

## Abstract

Participation in community club-based sport delivers significant physical, social, and psychological health benefits, yet the cost to play is consistently identified as one of the most common barriers to participation across all age groups. Existing data have largely been limited to registration fees paid to sport organisations, leaving the true financial burden on individuals and families poorly understood. This study investigated the comprehensive costs associated with community sport participation across Australia. An online survey was conducted in collaboration with the Australian Sports Commission between October and December 2024, with 5,533 sports participants, including 2,457 children and 3,076 adults providing valid cost data. Costs were examined across sport type, sport size, socioeconomic status, and residential location, using descriptive statistics and ANOVA, with mean and medians costs examined. The average total annual cost to participate in community-level sport was $4,567 for children and $2,894 for adults—substantially higher than previously reported figures, which excluded costs such as equipment, coaching, travel and loss of parental income due to sport commitment/travel. Entry-level costs alone averaged $1,161 for children and $1,200 for adults. Costs varied considerably by sport, with individual sports more expensive than team sports for both children and adults. The overall total cost for children to play sport were similar for those living in major cities compared to regional/remote areas. However, there were regional differences with children in regional and remote areas facing significantly higher travel and accommodation costs than those in major cities, and children in cities paying more for coaching and registration fees. Two thirds of all participants (67%) reported that cost was a limiting factor in their participation decision, rising to 78% for children, and was disproportionately reported among persons with a disability, CALD participants, and those in the most disadvantaged areas. These findings provide comprehensive evidence to date of the true cost of community sport participation in Australia, with significant implications for sport policy, funding, and sport's governing bodies to ensure sport remains accessible for all Australians.

## Introduction

Participation in community club-based sport is popular especially for children. Across countries, participation in organised sport is consistently high for children and adolescents before declining considerably through late adolescence and throughout adulthood (1–3). In Australia over half of all children aged 5–15 participate regularly in organised sport (1, 4). Similar patterns of participation are reported internationally, with the United States reporting over 66% of children and adolescents aged 9–13 years participating in organised sport (5).

Whilst participation in organised community sport is popular, there are many different factors influencing participation. Consistently, the cost to play sport is reported as a major barrier to participation (6–9). Globally, the two most commonly reported barriers for children's participation in sport is cost and time (10). However, the cost to play is a major barrier across all ages (8, 11).

The costs associated with participation in sport are both a barrier to participation and a major contributing factor to dropout. Further, low socioeconomic households and communities are much less likely to participate at both the community level, and subsequently representative levels of participation (7, 9, 12). Rising costs of living have further intensified financial challenges and increased financial pressures, leading individuals to modify their physical activity behaviours and often reducing participation in organised sport (8).

The costs associated with playing sport are quite different internationally. For example, in the United States mass sport participation is privately organised through community organisations rather than governed by national sports organisations. This leads to a pay-to-play model with substantial burden on the individual sport participants and their families. By contrast, in Europe and specifically Northern Europe, participation is largely club-based and governments heavily subsidise community sport (8). In Australia, local community sports clubs are not-for-profit organisations run by volunteers (13). As such participants generally pay a registration or membership fee. Further costs may be associated with equipment, competitions, coaching, apparel and travel, and these costs are not consistent across sports nor level of competition with costs at a community level of participation considerably lower than for players at a state, national or international levels (8). Costs differ according to activity with organised competitive sport associated with higher participation costs compared to many individual physical activities which can be undertaken at little or no cost (7).

There is limited data reporting the total or true cost to individuals and families to participate in community sport. Previous national studies such as AusPlay in Australia have reported on the expense paid to organisations to participate in sport, but these do not take into account the other associated costs. Reports for 2022–2023 indicated that for children the average annual costs paid to organisations or venues for children's sport participation were $899 for ages 0–4, $1,382 for ages 5–8, $1,450 for ages 9–11, and $1,760 for ages 12–14 (8).

Globally there is consistent reporting that the costs associated with playing community sport have risen in recent years. In Australia, costs rose considerably, for children the average cost in 2022–2023 being AUD $1,369 rising from AUD $685 in 2018–2019. Similarly, costs associated with participation rose for adults from AUD $796 in 2018–2019 to AUD $1,304 in 2022–2023 (14). A recent study in Northern Ireland reported that 30% of families were unable to afford the equipment and uniforms that their children needed to play sport (15).

To assess how sport participation costs have tracked against broader economic conditions, cost increases were compared against changes in Australia's Consumer Price Index (CPI) over the same period (2018–2019 to 2022–2023) (16). Percentage change for each measure—CPI, children's costs, and adult costs—was calculated as the difference between the 2022–2023 and 2018–2019 figures, expressed as a proportion of the 2018–2019 baseline. The relative rate of increase (reported as sport costs rising at “four to seven times” the rate of inflation) was then calculated by dividing each sport cost growth rate by the CPI growth rate over the same period. To illustrate the scale of this divergence in dollar terms, a counterfactual cost was also calculated: applying the observed CPI growth rate, rather than the actual observed increase, to the 2018–2019 baseline cost, to estimate what the 2022–2023 cost would have been had it risen only in line with inflation.

These cost increases are substantially greater than general inflation over the same period. Australia's Consumer Price Index (ABS data) rose from approximately 79.3 in 2018–2019 to approximately 90.9 in 2022–2023, representing a cumulative increase of roughly 14%–15% over that period. By comparison, children's sport participation costs nearly doubled (+100%), and adult costs rose by approximately 64%, meaning sport costs rose at four to seven times the rate of general inflation (16). To put this in context, if children's sport costs had risen in line with CPI, the average cost in 2022–2023 would have been approximately $785 rather than $1,369. This divergence suggests that cost pressures in community sport are not simply a reflection of broader economic conditions, but are driven by structural factors consistent with evidence around the proliferation and professionalisation of youth sport (17). These above-inflation increases compound the financial burden on families and amplify barriers to participation, particularly for those on lower incomes.

Decisions about sport participation are shaped by both supply-side and demand-side factors. On the demand side, individuals and families may weigh the perceived benefits of participation—physical, social, and psychological (18)—against the total costs involved, including fees, equipment, travel, and time (9, 12). On the supply side, the structure and pricing of sport organisations, the availability of subsidies, and the accessibility of facilities all influence what is available and at what cost (8). When costs rise or perceived value declines, demand-side responses include reducing the number of sports played, switching to lower-cost alternatives, or withdrawing from organised sport entirely (7). Understanding the full cost of participation is therefore essential for assessing the equitable access to sport, considering an intersectional lens for those facing more than one financial barrier to participation, and informing both policy and organisational decision-making.

There are increasing costs associated with participation in community sport, and cost is a major barrier to both participation and retention. However, research on the cost to play has largely focused on the cost of registration or fee paid to the organising body and not the total costs associated with participation. Therefore, the aim of this study was to investigate the comprehensive costs associated with participation in community sport across Australia.

## Methods

In collaboration with the Australian Sports Commission (ASC), the research team developed a survey to investigate the cost to play sport in Australia. The focus of the study was on community club-based sport, however the survey was not limited to only those local community players and included those playing regional, State and National or International competitions.

An online survey using Qualtrics was open to respondents from October to 31st December 2024. The survey was advertised through the researcher team and ASC media and social media as well as direct communication from the ASC to National Sport Organisations (NSOs) and National Sport Organisations for people with a disability (NSODs) encouraging their distribution to registered players, and a marketing campaign. The email to sports organisations and social media posts contained a link to an online survey and indicated that the study was investigating the costs for participants to play sport in Australia. The survey was open to Australians who were registered sport participants.

### Measures

Sport participation variables included the sport type, level, frequency, and duration of participation. Sport levels included: community level, regional level, state, national, and international and were combined into community level (community level, regional level) and state or above (state, national, and international). Additional analyses were conducted for community level and including only the entry level costs. These were defined by the research team together with the ASC and included registration/membership fees, uniform/clothing and equipment.

Sports were classified as individual or team sports using the AusPlay categories. The size of the sport organisation was categorised into small, medium, and large according to the ASC predefined categories.

The cost of participation variables included the specific costs (registration fees, court hire, uniform, equipment, coaching, tournaments/competition etc), perceived value of the cost of registration fees, and changes to cost of registration fees. Reported costs are in AUD. Individuals were asked to estimate the cost in $AUD for each item for them to play their sport within the past 12 months/season. If individuals were registered to play more than one sport, they were asked to report the costs associated with their favourite sport.

Demographic variables included the participants date of birth, sex, Aboriginal and/or Torres Strait Islander status, language spoken at home, disability status, sexual orientation (adults only), household structure, highest education, employment, and postcode of residence. Individuals who were aged under 18 years of age were asked to provide parental consent. Those aged under 13 years were asked to complete the survey with a parent.

Postcode of residence was used to classify socioeconomic status according to the Socioeconomic Index for Area (SEIFA) (19), specifically the Index of Relative Socio-economic Advantage and Disadvantage (IRSAD) which ranks areas in Australia according to relative advantage and disadvantage. Postcode level SEIFA percentiles were categorised into quartiles, with the lowest 25% being classified as the most disadvantaged (1st quartile) and the highest 25% classified as the least disadvantaged (4th quartile). Postcode was also used to categorise major cities, regional and remote areas according to the Accessibility and Remoteness Index of Australia (ARIA+) (20).

Previous research studies and literature and consultation with the Australian Sports Commission was used to inform the content of the survey. The survey was piloted with sport participants.

### Data analysis

Descriptive statistics, including frequencies and proportions, were calculated for all demographic characteristics. Missing data was excluded from each analysis. We calculated the mean and 95% confidence intervals for the annual cost to participate in sport and used analysis of variance (ANOVA) to compare the mean costs across subgroups. Chi-squared tests were used to compare categorical variables across subgroups. As the data was skewed, we also calculated median with the interquartile range. All analyses were conducted in SAS Enterprise Guide 9.4 (SAS Institute, Cary, NC, USA).

The main comparisons were calculated separately for children and adults and by:

- Sport type (individual, team)
- Sport size (small, medium, large)
- SEIF quartile
- Location (major cities, regional/remote)

When interpreting the results, caution should be given to those categorical responses with small sample sizes, as the estimates may not be accurate.

### Ethics

Ethics approval was granted by the Federation University Australia Human Research Ethics Committee (2024/179). Participants who completed the survey and provided their contact details were eligible to enter a draw to win one of five $100 gift vouchers.

## Results

### Survey participants

Of the 9,529 people who started the survey, a total of 5,533 (58%) people reported participation in an included sport and provided valid cost data. Of these, 2,457 (44%) were children aged under 18 years and 3,076 (56%) were adults aged 18 years and over.

Over half of respondents were female (60%) compared to 39% male and 1% other or prefer not to say. A small proportion of respondents identified as Aboriginal and/or Torres Strait Islander (3%) or spoke a language other than English (7%).

Most respondents did not report a disability (85%). However, 4% of respondents reported a social/emotional disability, 3% reported a chronic health condition, and 3% reported a physical disability.

The most common employment type was full-time (38%), with another 11% of respondents working part-time, and 20% of respondents reporting home duties. More than one third of respondents lived in the most advantaged quartile (38%), compared to only 15% from the most disadvantaged quartile.

All States and Territories were represented with approximately one third of respondents living in NSW (29%), one fifth living in Victoria (21%), and another fifth living in Queensland (18%). Most respondents lived in major cities (61%).

Whilst this study included costs separately for those participating in community-level sport and state or above level the focus of this paper are community-level participants.

### Participation in sport

#### Children

Of the children who participated at the community level, 73% participated in a team sport, and 27% in an individual sport. Three quarters of children participated in a large sport (73%), 20% a medium sport and 7% a small sport.

Children had played the sport on average age for 5.8 years. They participated on average in 3.2 sessions per week and more often for team-based sport (3.3 sessions) compared to individual sports (2.7 sessions).

A total of 67 different sports were represented with the main sports being Football (soccer; *n* = 342, 21%), Basketball (*n* = 202, 13%), Netball (*n* = 140, 9%), Swimming (*n* = 128, 8%), and Australian Football (AF; *n* = 115, 7%).

#### Adults

Of the adults who participated at the community level, 48% participated in a team sport, and 52% in an individual sport. Nearly half of adults participated in a large sport (48%), 37% a medium sport and 15% a small sport.

Adults had played the sport on average for 18.3 years. They participated on average in 2.8 sessions per week and similarly for team sports (2.7 sessions) compared to individual sports (2.8 sessions).

Overall, 77 different sports were represented with the main sports being Bowls (*n* = 300, 14%), Hockey (*n* = 211, 10%), Football (Soccer; *n* = 154, 7%), Croquet (*n* = 142, 7%), and Netball (*n* = 122, 6%).

### The cost to play sport

#### Children

The average cost to participate in community level sport for children was $4,567. Entry level costs included registration/membership fees, uniform/clothing and equipment. The average entry-level cost to play sport for children was $1,161. This consisted of on average $307 for uniform/clothing (including shoes), $474 for registration/membership fees, and $379 for equipment. Entry level costs varied across sports, with the lowest entry level costs reported for Volleyball ($266) and Softball ($271) compared to highest entry level costs associated with Equestrian ($8,707) and Gymnastics ($1,715).

The average total cost of play small ($5,054), medium ($4,574), and large sports ($4,520) was similar. However, compared to large sports, participation in small sports cost an additional $385 for coaching (*p* < 0.001) and an additional $143 for equipment (*p* < 0.01).

Overall, there were no differences in the total cost of sport participation across States and Territories. However, the cost of competition travel differed with New South Wales reporting the highest average travel cost ($733) and the Australian Capital Territory the lowest travel cost ($395).

The costs to play were similar across SEIFA quartiles. However, those most disadvantaged had higher expenses associated with tournament/competition accommodation ($716) compared to most advantaged ($386) (*p* < 0.001) as well as higher costs associated with travel ($745 vs. $447) (*p* < 0.001). A similar pattern was evident across location with much higher costs associated with travel for regional/remote participants ($790) compared to those living in major cities ($428) (*p* < 0.001) and tournament/competition accommodation ($714 vs. $386) (*p* < 0.001).

**Figure 1:**
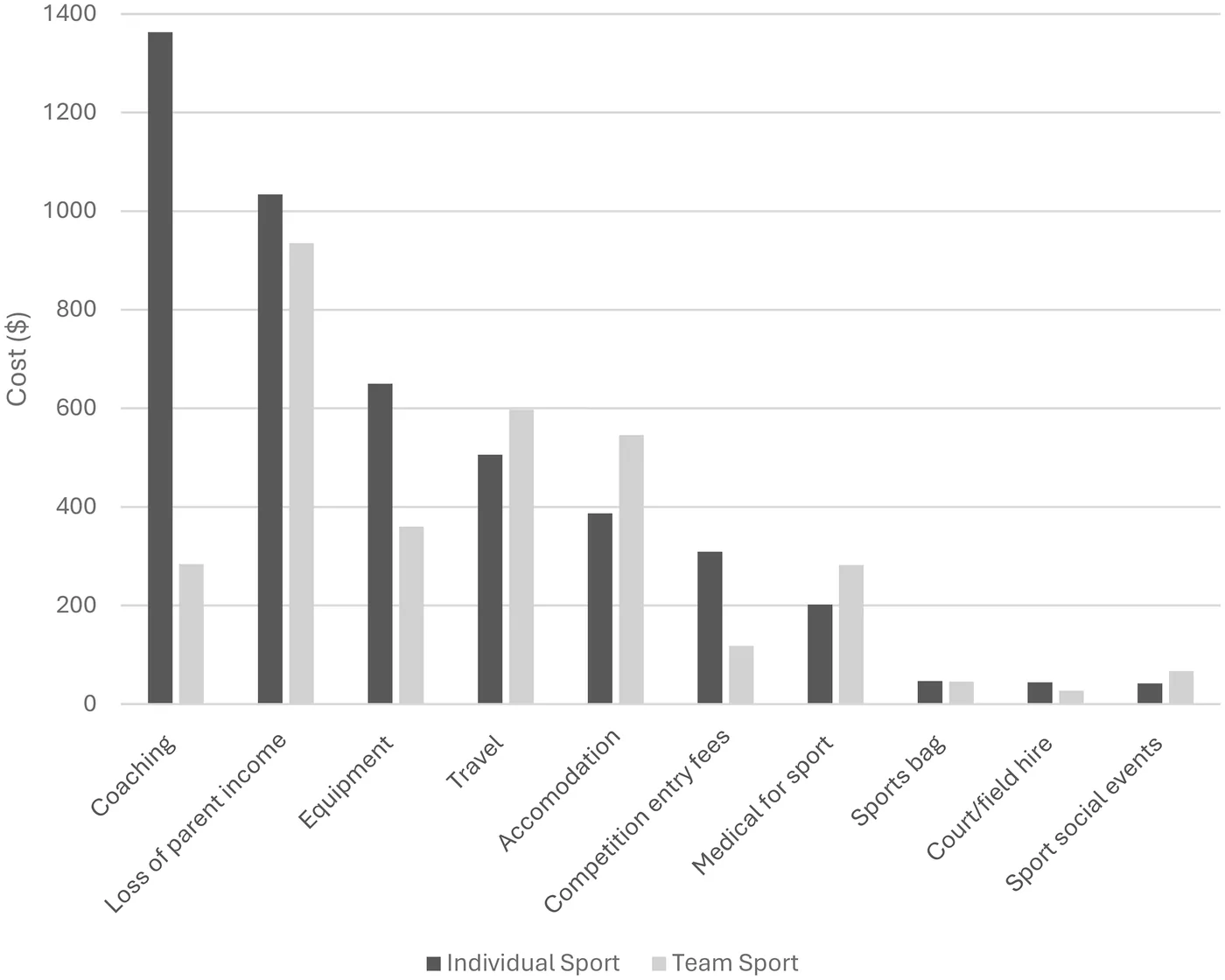
Main costs of community level sport participation by location for children.

Individual sports had a higher average expense ($5,401) compared to team-based sports ($4,260) (*p* < 0.01) (Table 1 and Figure 1), with higher costs associated with coaching ($1,363 vs. $284) (*p* < 0.001), equipment ($650 vs. $360) (*p* < 0.001) and tournament/competition fees ($309 vs. $118) (*p* < 0.001). However, team sports participants had higher club registration fees ($621 vs. $476) (*p* < 0.001) and fees associated tournament/competition accommodation ($546 vs. $387) (*p* < 0.01).

**Table 1:** Cost of sport participation by sport type for children.

Cost item	Individual sport		Team sport		*p*-value
	Mean ($)	(95% CIs) ($)	Mean ($)	(95% CIs) ($)	
Total (*n* = 1,620)	5,401	(4,702, 6,099)	4,260	(3,915, 4,604)	<0.01
Clothing/uniform	356	(308, 404)	390	(371, 410)	0.11
Club registration/membership fee	476	(401, 551)	621	(579, 662)	<0.001
Coaching	1,363	(1,159, 1,568)	284	(234, 334)	<0.001
Court/field hire	44	(27, 60)	27	(21, 34)	0.03
Equipment	650	(435, 865)	360	(312, 407)	<0.001
Sports bag	47	(41, 54)	46	(42, 49)	0.64
Loss of parent/caregiver income due to sport commitment/travel	1,034	(698, 1,370)	935	(752, 1,118)	0.59
Medical for sport	202	(121, 284)	282	(188, 376)	0.34
Player insurance	32	(20, 43)	32	(25, 39)	0.89
Sport social events	42	(35, 50)	67	(61, 73)	<0.001
Tournament/competition accommodation	387	(302, 472)	546	(480, 612)	<0.01
Tournament/competition entry/spectator fees	309	(241, 378)	118	(95, 141)	<0.001
Travel	506	(407, 606)	597	(534, 660)	0.14

**Figure 2:**
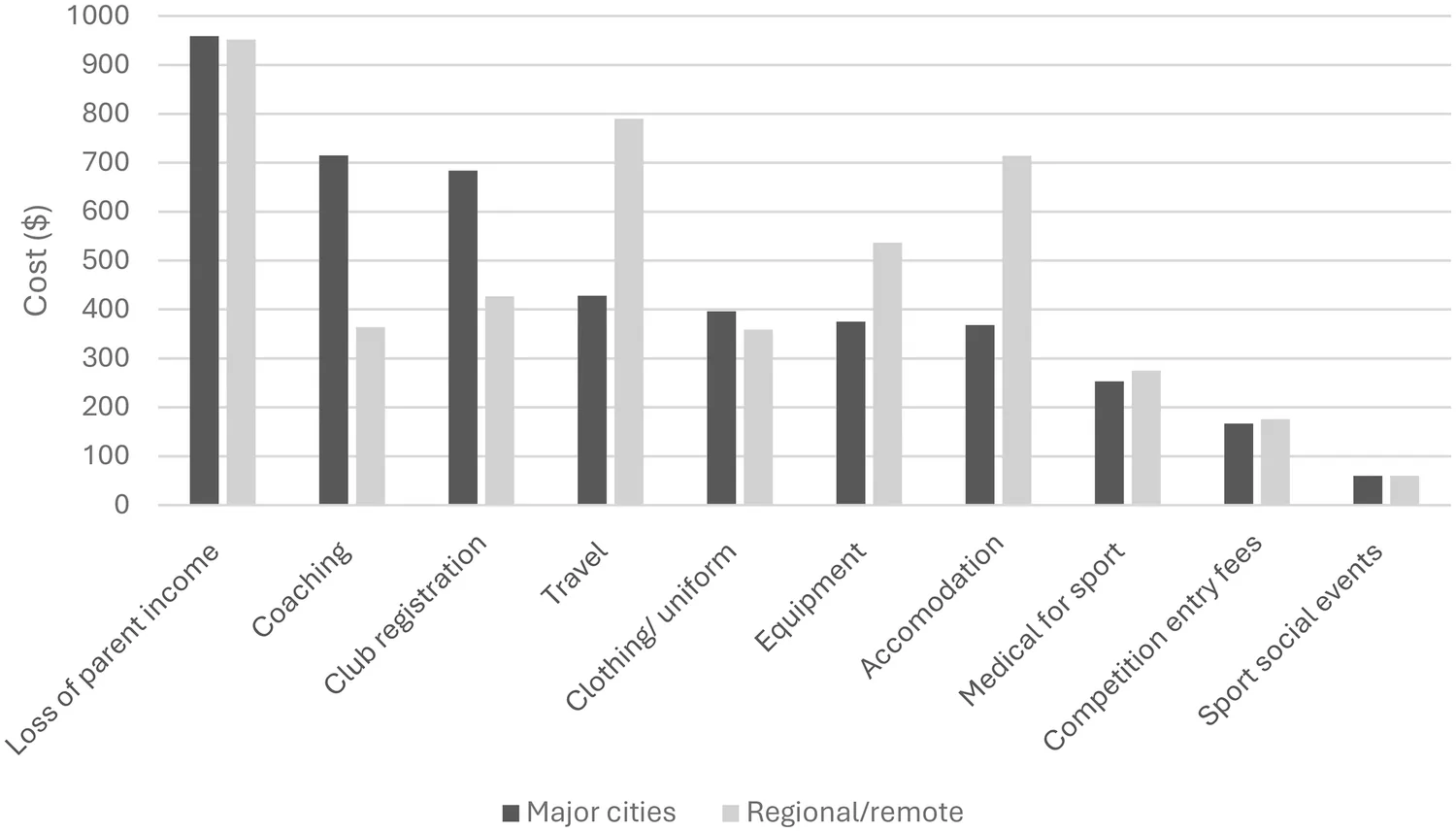
Main costs of community level sport participation by location for children.

Children living in major cities and regional/remote areas had similar average total costs of sport participation ($4,465 vs. $4,727). However, children living in major cities reported paying an additional $351 for coaching (*p* < 0.001) and an additional $257 for club registration/membership fees (*p* < 0.001) (Table 2, Figure 2). In contrast, children living in regional/remote areas reported paying an additional $362 for competition travel (*p* < 0.001) and an additional $346 for tournament/competition accommodation (*p* < 0.001). The main costs of community level sport participation by location is provided in Figure 1 (*n* = 1,620).

**Table 2:** Cost of sport participation by remoteness for children.

Cost item	Major cities		Regional/remote		*p*-value
	Mean ($)	(95% CIs) ($)	Mean ($)	(95% CIs) ($)	
Total (*n* = 1,620)	4,465	(4,069, 4,861)	4,727	(4,208, 5,246)	0.72
Clothing/uniform	396	(372, 421)	359	(329, 389)	0.17
Club registration/membership fee	684	(632, 736)	427	(381, 473)	<0.001
Coaching	715	(613, 818)	364	(281, 447)	<0.001
Court/field hire	33	(24, 42)	30	(21, 40)	0.62
Equipment	375	(311, 439)	537	(395, 679)	0.07
Sports bag	44	(40, 48)	50	(44, 55)	0.19
Loss of parent/caregiver income due to sport commitment/travel	959	(729, 1,188)	952	(745, 1,159)	0.78
Medical for sport	253	(198, 309)	275	(112, 439)	0.87
Player insurance	26	(20, 31)	42	(30, 55)	0.02
Sport social events	60	(54, 65)	60	(52, 68)	0.34
Tournament/competition accommodation	368	(312, 424)	714	(610, 817)	<0.001
Tournament/competition entry/spectator fees	167	(140, 195)	176	(127, 225)	0.66
Travel	428	(370, 486)	790	(690, 891)	<0.001

The cost of sport participation varied considerably across sports (Table 3). The highest costing sports included Water Polo ($15,694), Equestrian ($13,947) and BMX/mountain biking ($11,699). The sports with the lowest costs included Taekwondo ($1,127), Softball ($1,643) and Athletics ($1,970).

**Table 3:** Total cost of sport participation by sport for children.

Sport	*n* *	Mean ($)	(95% CIs) ($)
Athletics	27	1,970	(1,227, 2,713)
Australian Football (AFL)	115	3,896	(3,184, 4,608)
Baseball	38	4,815	(3,284, 6,346)
Basketball	202	6,369	(5,490, 7,247)
Calisthenics	5	3,082	(381, 5,784)
Cricket	76	4,931	(3,822, 6,040)
BMX, Mountain Bike	8	11,689	(3,589, 19,789)
Dance Sport	50	4,541	(3,282, 5,800)
Equestrian	16	13,947	(7,113, 20,781)
Football (Soccer)	342	5,622	(5,026, 6,217)
Golf	6	4,286	(856, 7,715)
Gymnastics	76	6,416	(4,973, 7,858)
Hockey	86	6,043	(4,766, 7,320)
Ice Hockey	5	5,677	(701, 10,653)
Karate	11	3,346	(1,369, 5,323)
Netball	140	7,543	(6,293, 8,792)
Roller Sports	10	2,518	(957, 4,079)
Rugby League	40	4,903	(3,384, 6,423)
Rugby Union	36	4,759	(3,205, 6,314)
Softball	19	1,643	(904, 2,382)
Surf Life Saving	10	10,239	(3,893, 16,585)
Swimming	128	8,151	(6,739, 9,563)
Taekwondo	7	1,127	(292, 1,962)
Tennis	41	7,320	(5,079, 9,561)
Touch	48	4,981	(3,572, 6,390)
Volleyball	14	3,184	(1,516, 4,852)
Water Polo	12	15,694	(6,814, 24,573)

* Includes those with 5 or more responses.

#### Adults

The average cost of participation in community level sport for adults was $2,894. The average entry level cost to participate in sport was $1,200. This consisted of an average cost of $285 for uniform/clothing (including shoes), $361 for registration/membership fees, and $554 for equipment. Entry level costs varied across sports, with the lowest entry level costs reported for Rugby League ($261) and Wrestling ($269). Sports with the highest entry levels costs were Shooting ($7,062) and Equestrian ($6,127).

Participation costs for small sports were higher ($3,935) than costs for medium ($3,001) and large sports ($2,482) (*p* < 0.001). Compared to large sports, small sports cost an additional $592 for equipment (*p* < 0.001), an additional $317 for coaching (*p* < 0.001) and an additional $234 for court/field hire (*p* < 0.001).

Overall, there were no differences in the average total cost of sport participation for adults across States and Territories. There were some differences across States and Territories for specific costs including equipment, with the highest costs being reported in the Northern Territory ($1,694) and the lowest cost in Tasmania ($322).

There was no difference in cost by location with similar costs for those living in major cities compared to regional/remote locations.

Overall, there were no differences in the total cost of sport participation for adults living in disadvantaged areas compared to those living in advantaged areas.

**Figure 3:**
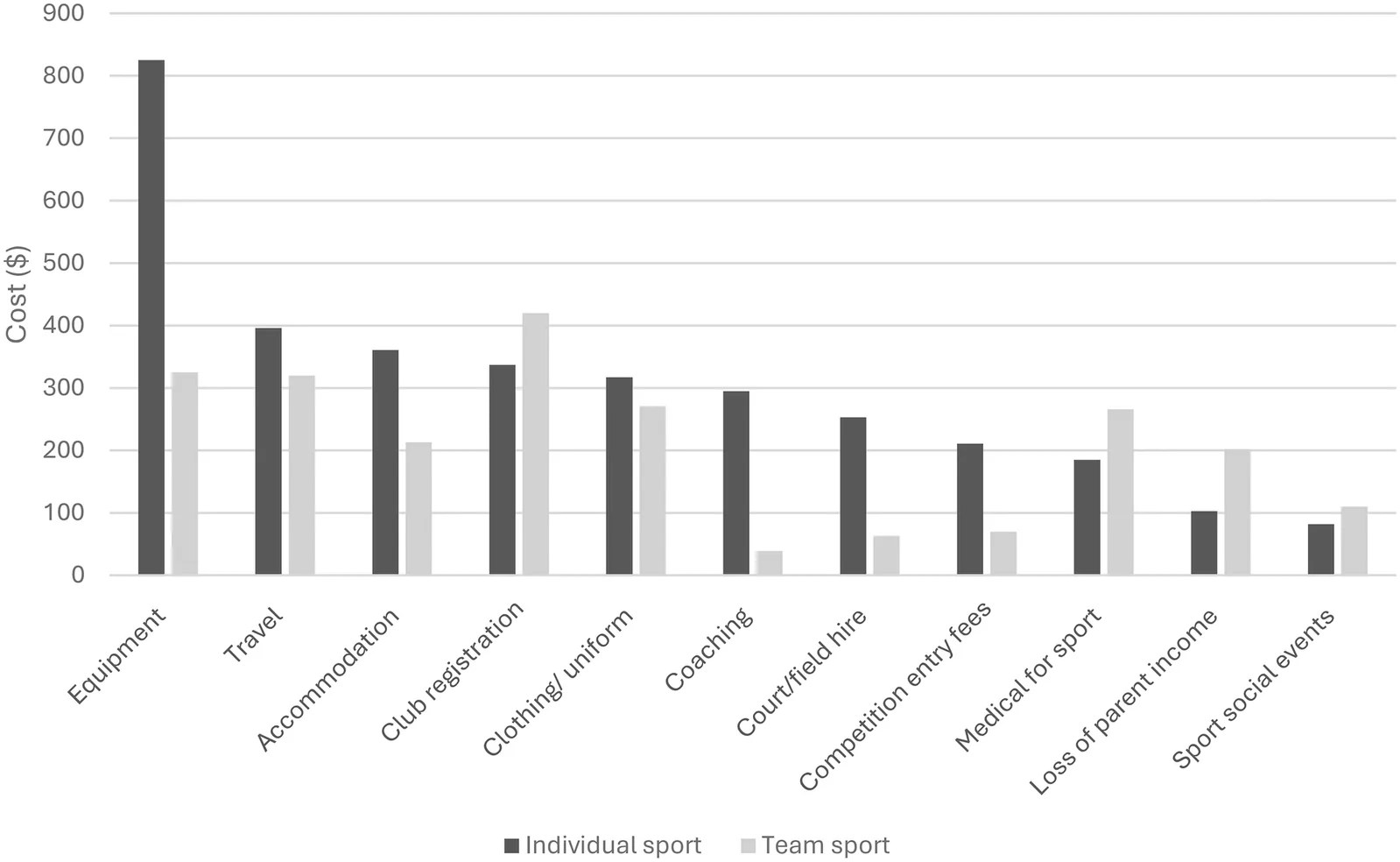
Main costs of community level sport participation by sport type for adults.

Adults participating in individual sports had higher costs ($3,390) compared to team sports ($2346) (*p* < 0.001) and had higher costs associated with clothing/uniform, coaching, court/field hire, equipment, tournament/competition entry fees and travel (Table 4) (Figure 3).

**Table 4:** Cost of sport participation by sport type for adults.

Cost item	Individual sport		Team sport		*p*-value
	Mean ($)	(95% CIs) ($)	Mean ($)	(95% CIs) ($)	
Total (*n* = 2,134)	3,390	(3,123, 3,658)	2,346	(2,165, 2,528)	<0.001
Clothing/uniform	317	(296, 338)	271	(259, 284)	<0.001
Club registration/membership fee	337	(300, 375)	420	(397, 443)	<0.001
Coaching	295	(243, 347)	39	(27, 51)	<0.001
Court/field hire	253	(202, 304)	63	(48, 79)	<0.001
Equipment	825	(694, 956)	325	(295, 354)	<0.001
Sports bag	43	(38, 47)	40	(37, 44)	0.44
Loss of parent/caregiver income due to sport commitment/travel	103	(47, 159)	202	(89, 314)	0.11
Medical for sport	185	(152, 218)	266	(227, 306)	<0.01
Player insurance	26	(19, 32)	49	(42, 55)	<0.001
Sport social events	82	(71, 92)	110	(99, 120)	<0.001
Tournament/competition accommodation	361	(311, 412)	213	(162, 264)	<0.001
Tournament/competition entry/spectator fees	211	(184, 237)	70	(60, 80)	<0.001
Travel	396	(338, 454)	320	(277, 363)	0.04

Overall, there was no evidence of a difference in the average total cost of sport participation across socioeconomic status quartiles. There was also no different in total average cost for adults living in major cities compared to regional/remote areas.

The cost of sport participation varied considerably across sports (Table 5). The highest costing sports included Motor Sports ($14,551), Weightlifting ($8,923), and Equestrian ($8,580). The sports with the lowest costs included Tenpin bowling ($509), Rugby League ($660), and Flying Disc ($812).

**Table 5:** Total cost of sport participation by sport for adults.

Sport	*n* *	Mean ($)	(95% CIs) ($)
Archery	11	2,356	(964, 3,749)
Athletics	25	3,469	(2,109, 4,828)
Australian Football (AFL)	65	2,171	(1,643, 2,699)
Badminton	11	2,732	(1,117, 4,346)
Baseball	49	1,646	(1,185, 2,107)
Basketball	64	2,778	(2,097, 3,459)
BMX, Mountain Bike	31	7,973	(5,167, 10,780)
Bowls	300	1,656	(1,469, 1,843)
Canoeing	6	3,466	(693, 6,240)
Cricket	63	1,443	(1,087, 1,800)
Croquet	142	1,838	(1,536, 2,141)
Dance Sport	6	2,088	(417, 3,759)
Dragon Boat	54	1,338	(981, 1,695)
Equestrian	29	8,580	(5,457, 11,703)
Fencing	15	2,156	(1,065, 3,248)
Flying Disc	5	812	(100, 1,524)
Football (Soccer)	154	5,268	(4,436, 6,100)
Golf	82	5,075	(3,977, 6,174)
Gymnastics	14	2,204	(1,049, 3,359)
Hockey	211	1,811	(1,567, 2,055)
Ice Hockey	51	3,268	(2,371, 4,165)
Judo	5	2,602	(321, 4,882)
Karate	7	2,694	(698, 4,689)
Lacrosse	9	1,390	(482, 2,298)
Motor Sport	5	14,551	(1,797, 27,306)
Netball	122	2,231	(1,835, 2,627)
Orienteering	19	2,955	(1,627, 4,284)
Pickleball	34	2,831	(1,879, 3,783)
Roller Sports	52	5,474	(3,986, 6,962)
Rowing	14	3,733	(1,778, 5,689)
Rugby League	11	660	(270, 1,049)
Rugby Union	21	3,052	(1,747, 4,357)
Running/jogging	6	1,972	(394, 3,550)
Sailing	11	4,335	(1,773, 6,897)
Shooting	22	6,987	(4,067, 9,906)
Softball	48	3,515	(2,521, 4,510)
Squash	14	3,159	(1,504, 4,814)
Surf Life Saving	7	2,912	(755, 5,070)
Swimming	51	4,006	(2,906, 5,105)
Table Tennis	16	4,189	(2,136, 6,241)
Taekwondo	6	2,218	(443, 3,993)
Tennis	83	1,811	(1,421, 2,200)
Tenpin Bowling	6	509	(102, 916)
Touch	21	1,012	(579, 1,445)
Triathlon	24	7,156	(4,293, 10,018)
Volleyball	31	2,301	(1,491, 3,111)
Water Polo	21	3,890	(2,226, 5,554)
Weightlifting	30	8,923	(5,730, 12,117)
Wrestling	7	5,230	(1,356, 9,104)

* Includes those with 5 or more responses.

## Registration fees

Over a third (38%) of participants felt that the registration fees were *about right*. Children were more likely than adults to report that their registration fees were *too high* (67% vs. 49%) (*p* < 0.001).

There were some demographic groups who felt that their registration fees were *far too high**.*** This included those with a disability (29%) (*p* = 0.01), and CALD participants (31%) (*p* < 0.01).

Team sport participants were more likely to report feeling that their registration fees were *too high* compared to individual sport participants (68% vs. 39%) (*p* < 0.001). Large and medium sport participants were more likely to report feeling that their registration fees were *too high* compared to small sport participants (58% vs. 46%) (*p* < 0.01).

The feelings about registration fees were similar according to SEIFA quartile (*p* = 0.18).

Participants living in regional/remote areas were less likely to report feeling that their registration fees were *too high* compared to participants living in major cities (52% vs. 59%) (*p* < 0.01).

## Cost as a limiting factor

Overall, 67% reported that cost was a limiting factor in their decision to participate in sport, and this was more common for children (78%) compared to adults (59%) (*p* < 0.001); for persons with a disability (75%) compared to those without a disability (66%) (*p* < 0.001); for CALD participants compared to non-CALD participants (78% vs. 66%) (*p* < 0.001); team sport participants compared to individual sport participants (73% vs. 59%) (*p* < 0.001); small sport participants compared to large sport participants (70% vs. 67%) (*p* = 0.03); and participants living in the most disadvantaged areas compared to participants living in the most advantaged areas (72% vs. 63%) (*p* < 0.001).

There were no significant differences reporting cost as a limiting factor across major cities and regional/remote areas (*p* = 0.38).

## Registration fee increase

There were some demographic groups that reported that their registration/membership fees had increased since last year/season. These were more likely to be: children compared to adults (66% vs. 56%) (*p* < 0.001); team sport participants compared to individual sport participants (67% vs. 52%) (*p* < 0.001); large and medium sport participants compared to small sport participants (large: 62% medium: 63%; small: 47%) (*p* < 0.001); and participants living in major cities compared to those living in regional/remote areas (63% vs. 57%) (*p* < 0.001). There was no difference according to SES.

## Discussion

The study clearly demonstrates that the total cost associated with playing community sport in Australia is high, especially for children, and those costs continue to increase at a rate higher than inflation. On average it amounts to $4,500 for a child to play one sport for a season or year and over $2,800 for adults. These are much higher than previously reported in other reports with amounts between $1,450 and $1,760 for children aged 9–14 years (8). However, these previous figures only take into account costs paid to an organisation to play sport and do not include other costs required for participation such as equipment and uniforms. The minimum viable cost at the entry level to play sport are also considerable, with an average $1,200 for both children and adults. The current study provides a comprehensive understanding of the total costs associated with playing community sport in Australia.

This study provides further breakdowns of costs by sport type and size and residential location. It is clear that the costs to play sport are quite different according to the particular sport, for individual compared to team and for those living regionally/remotely compared to living in metropolitan cities.

Some sports are reasonably affordable whilst other are significantly more expensive. Sports with specialised equipment such as motor bikes, BMX bikes, horses for equestrian were generally much more expensive than sports such as taekwondo, softball, rugby league and athletics. It is not clear how the decision to play a particular sport is impacted by the associated costs with a particular sport, and whether or not rising costs to play sport will influence individuals and family's choices of sports and activities.

Individual sports were generally more expensive to play, mainly due to higher expenses for coaching, equipment and court/field hire, compared to those playing team sports where coaches are generally un-paid volunteers. Many individual sports like tennis and golf require participants to own their own equipment, which contributes to costs, where equipment is often cheaper, and often owned by the club for many team sports such as netball, football and soccer.

Travel was an expense that differed significantly by participant location with children in regional/remote areas on average spending $790 for travel and a further $714 for accommodation compared to city living children paying on average $428 for travel and $368 for accommodation. Travel costs were more evident amongst the children compared to adults as children are more likely to play more competitions and tournaments which require additional travel. Regional and remote living persons do not often have access to public transport, combined with the distance required to travel to participate, accommodation is often a necessary expense not experienced in cities (21).

The costs were similar across SEIFA quartiles, meaning that disadvantaged families are spending similar amounts of their household budget on sport as those in less disadvantaged areas. However, it must be acknowledged that this does not indicate equivalency in affordability as those disadvantaged families are proportionally spending more of their money to play community sport.

Further, the composition of those costs differ in important ways, particularly when there were compounding factors influencing costs, such as living in regional areas. Families in more disadvantaged and regional or remote areas faced significantly higher expenses for travel and tournament accommodation—costs that are less discretionary and harder to avoid than, say, coaching fees. This pattern suggests that the financial burden on disadvantaged and regionally located participants is not captured by total cost figures alone, and that targeted supports such as travel subsidies or regional competition structures may be warranted. Children living in major cities, by contrast, faced higher coaching and club registration costs, which is consistent with evidence that metropolitan clubs are more likely to be operating at a financial deficit and reliant on registration fee income to remain sustainable.^1^

Two thirds of children and a half of the adults thought that registration fees were too high and those living in cities were more likely to report this too. Furthermore, those with a disability and CALD participants were more likely to report that registration fees were far too high, but with no difference across SEIFA quartiles. This suggests that further work could be done across the sector to exemplify the value offered for the price of registration fees, and consider how subsidised approaches have improved registration fee affordability for eligible participants (e.g., low SEIFA families), and how this eligibility may be considered for other target groups that feel registration fees are cost prohibitive, including those with disability and CALD communities.

## Cost as a limiting factor

The finding that two thirds of all participants reported cost as a limiting factor in their decision to participate in sport is striking, and particularly so for children (78%). However, given that this study included only current sport participants, this value is likely an underestimation of the impact of cost as a barrier to participation, as it does not include those who may have dropped out or never participated due to financial constraints. This is consistent with cost being one of the most commonly cited barriers to sport participation in the literature (6, 7, 10). That children are more affected than adults is not surprising given that participation decisions are made by families managing multiple competing financial demands, and that children's sport is often associated with more intensive competition schedules and associated costs. The higher rates reported among persons with a disability (75%), CALD participants (78%), and those in the most disadvantaged areas (72%) highlight that cost is not a uniform barrier—it falls most heavily on those already facing the greatest barriers to participation. Team sport participants were substantially more likely to report cost as a limiting factor than those in individual sports (73% vs. 59%), which may partly reflect the typically higher registration fees and structured competition costs associated with team sport. Together, these findings underscore that cost is not simply a background consideration but a real and active constraint on participation decisions for most sport participants in Australia. The total cost to play community sport and the affordability of playing sport can influence the choices of sports activities for children (12).

The choice to play community sport is bound by a range of factors including personal choice, accessibility, family familiarity and the affordability of playing sport. As cost of living pressures continue to affect Australian households, the cost to play will weigh heavily on individual and family decisions around sport participation. This may manifest in reduced participation overall, a reduction in the number of sports played simultaneously, or a shift toward lower-cost sporting options. In Northern Ireland, 35% of parents reported reduced spending on sport for children due to cost-of-living crisis, and 37% reported that they children had missed out on sport activity because of the costs involved (15). Further, low income households were more likely to report that their children missed out on playing sport in the previous year because of the costs involved (15).

The value of participation in sport is significant with improvements in a range of social, psychological and physical health benefits (18, 22). Sport is unique in its physical, cognitive and social opportunities and challenges within structured goal-directed contexts. Apart from the physical health benefits, participation in sport is consistently associated with higher self esteem, greater life satisfaction, and lower symptoms of depression and anxiety. Social outcomes associated with participation include enhanced belonging, prosocial behaviour and interpersonal skills (18). Consistently, these benefits are more evident through participation in team compared to individual sports due to the social nature of participation (18, 23).

How much the decisions around participation and dropout will be influenced by cost, relative to the perceived value of participation, remains to be seen as household budgets remain under pressure. It may be that decisions about participation in sport are not always made on the basis of health and wellbeing value alone, but are also shaped by social pressures and the desire to provide children with opportunities (9, 12). Families need to be able to appraise the costs and benefits of participation across different activities and ensure that these are in balance with their broader household resource demands (9). For many families the desire to have their children play sport and the drive to be a “good parent” often made them work hard at finding ways to manage the range of costs to participate (12).

## Limitations

Several limitations should be noted. The sample was recruited through National and State Sporting Organisations and their networks as well as the ASC media and social media, which may introduce self-selection bias. Respondents skewed toward female participants (60%) and toward those living in the most advantaged areas (38% in the highest SEIFA quartile compared to 15% in the lowest), which may mean that costs for the most disadvantaged families are underrepresented. As cost data were self-reported and retrospective, there is the possibility of recall error, particularly for costs accumulated across a season. The study focused on community-level participation, and findings may not generalise to higher levels of competition, though data on state-level and above participants were collected and will be reported separately. Finally, postcode-based SEIFA classification captures area-level disadvantage rather than individual household income, which is an approximation of socioeconomic status. Despite these limitations, the large national sample across 67 sports for children and 77 for adults provides a robust foundation for understanding the comprehensive costs of community sport participation in Australia. The survey was distributed through sports governing bodies and other social media platforms. It may be that highly engaged sport participants were more likely to complete the survey and therefore there may be self-selection bias. Further, there are limitations associated with the use of are-level socio-economic measure and not individual household income.

## Conclusion

This study provides the most comprehensive picture to date of the true cost of participating in community sport in Australia. The total annual cost—averaging over $4,500 for children and nearly $2,900 for adults—is considerably higher than previously reported figures, which have largely been limited to registration fees paid to organisations. Even entry-level costs of approximately $1,200 represent a meaningful financial commitment for many families. The cost burden has to be viewed through an intersectional lense: children, individuals with a disability, CALD participants, those in team sports, and those in regional and remote areas are most likely to report cost as a limiting factor. The composition of costs also varies significantly by sport type, sport size, and location in ways that have practical implications for both participants and sport administrators. As Australian households are under financial pressures, it is important that sport organisations, national bodies, and governments have access to accurate, comprehensive cost data to inform policy decisions, funding priorities, and fee-setting practices. Ensuring that the cost to play sport remains affordable and accessible will be central to maintaining participation rates and delivering the well-established health and social benefits that sport provides.

## Data Availability

The raw data supporting the conclusions of this article will be made available by the authors to qualified researchers, subject to ethical, legal and governance requirements.

## Footnotes

^1^Eime R, Westerbeek H, Owen K, Charity M, Keeping community sport a-oat: a national survey of the costs and financial strain of Australian community sports clubs. *Eur Sport Manag Q*, 2026.

## Appendix A: The median costs of sport participation at the community level for children and adults

**Table:**

	Children (<18 years) median (IQR)	Adults (18+ years) median (IQR)
Total	4,350 (1,550, 10,460)	2,430 (1,250, 5,963)
Clothing/uniform	350 (200, 680)	265 (150, 450)
Club registration/membership fee	300 (170, 700)	250 (130, 476)
Coaching	0 (0, 1,160)	0 (0, 60)
Court/field hire	0 (0, 0)	0 (0, 50)
Equipment	230 (80, 650)	250 (50, 750)
Sports bag	50 (0, 100)	0 (0, 80)
Loss of parent/caregiver income due to sport commitment/travel	0 (0, 1,000)	0 (0, 0)
Medical for sport	0 (0, 420)	20 (0, 400)
Player insurance	0 (0, 16)	0 (0, 45)
Sport social events	40 (0, 100)	50 (0, 120)
Tournament/competition accommodation	300 (0, 1,600)	0 (0, 900)
Tournament/competition entry/spectator fees	50 (0, 400)	50 (0, 300)
Travel	380 (0, 1,500)	200 (0, 1,000)
